# Qualitative evaluation of a hospital-inpatient service for children with medical complexity

**DOI:** 10.1136/bmjpo-2024-003101

**Published:** 2025-03-05

**Authors:** Swapnil Ghotane, Bethan Page, Rohana Ramachandran, Ingrid Wolfe, Lorna Katharine Fraser

**Affiliations:** 1 Department of Women and Children’s Health, King’s College London, London, UK; 2 Cicely Saunders Institute of Palliative Care, Policy and Rehabilitation, King’s College London, London, UK; 3 Evelina London Children’s Healthcare, Guy's and St Thomas' NHS Foundation Trust, London, UK

**Keywords:** Child Health, Qualitative research, Health services research, Caregivers

## Abstract

**Objective:**

To explore the experiences and perceptions of parents and professionals of a hospital-inpatient service for children with medical complexity (CMC).

**Design:**

Semi-structured qualitative interviews with parents of CMC and healthcare professionals from one hospital site in England. Data were analysed using thematic analysis.

**Findings:**

Nine parents and 15 healthcare professionals participated. Two overarching themes were developed: (1) The service is an anchor for families and professionals and (2) The service is not a panacea. Participants valued the single point of contact for families and professionals involved in the child’s care during hospital stays. Families felt heard, supported and involved in their child’s care with the holistic needs of the child and family centre stage. Unclear boundaries around the role of the service and limited capacity of the team were key challenges. Professionals and families described a cliff edge for many families post hospital discharge.

**Conclusion:**

A hospital-inpatient service for CMC can improve care coordination and discharge planning and help build strong relationships with parents so that they feel listened to and supported. Holistic services like this need clear boundaries and remits, as there is danger of ‘mission creep’. A hospital-inpatient service should not be seen as a panacea for meeting all the needs of CMC and their families. It is critical to understand how the service integrates with the wider health and care system.

**WHAT IS ALREADY KNOWN ON THIS TOPIC**

- Children with medical complexity (CMC) are increasing in number, and many have extensive unmet needs due to an inadequate and fragmented health and care system. There is a lack of evidence on the best models of care for these children and families.

**WHAT THIS STUDY ADDS**

- A dedicated hospital-inpatient service for CMC can play a unique and important role during hospital admissions, helping to coordinate care across specialties, supporting discharge arrangements and providing valuable support to parents. Services like this need clear boundaries around their role. Families may feel abandoned after discharge if there is no similar support in the community. Careful integration with community and primary care services is, therefore, required.

**HOW THIS STUDY MIGHT AFFECT RESEARCH, PRACTICE OR POLICY**

- Whole system approaches are needed for CMC to better meet the needs of these children and families. A dedicated inpatient holistic service can make a unique contribution to the multidisciplinary team supporting the child and family, but there are significant gaps in the wider system (eg, community and social care), which it has limited capacity to address. It is critical to have clear boundaries around the service and consider integration with the wider system.

## Introduction

 Children with medical complexity (CMC) are defined as having at least one chronic condition, technology dependence (eg, tube fed, long-term ventilation), multiple subspecialist involvement and substantial healthcare utilisation.^1 2^ Advances in acute medicine and medical technologies have improved survival.^3^ CMC are considered a ‘high-cost, high-need’ cohort.^4 5^ The provision and quality of care are often inadequate to meet the needs of these children and families, who struggle to navigate a complex and fragmented system.^6 7^ Parents balance their roles as caregiver, care coordinator and parent, contributing to stress, sleep disturbances and mental health issues.^8 9^ A key challenge is how to adapt health and care systems to better meet the needs of CMC and their families.

Integrated care models are considered a promising means of improving care for CMC; however, the evidence on what these models should look like is inconclusive.^10^ CMC often have long and frequent hospital admissions, and hospitals report concerns regarding wider patient flow due to increasing numbers of long-stay patients.^11^ A recent study of tertiary models of care for CMC in England concluded that there was a wide variety of models across the country, with limited evidence on effective and efficient models of care and little formal evaluation of services.^12^ Considering the current strain on the National Health Service^13^ and the growing population of CMC,^14^ it is vital that services and models of care are evaluated, in line with other high-income countries, such as Canada^15 16^ and the USA.^17 18^ This study aimed to explore the experiences and perceptions of parents and professionals of a hospital-inpatient service for CMC in England.

## Methods

### Study setting

**Figure 1:**
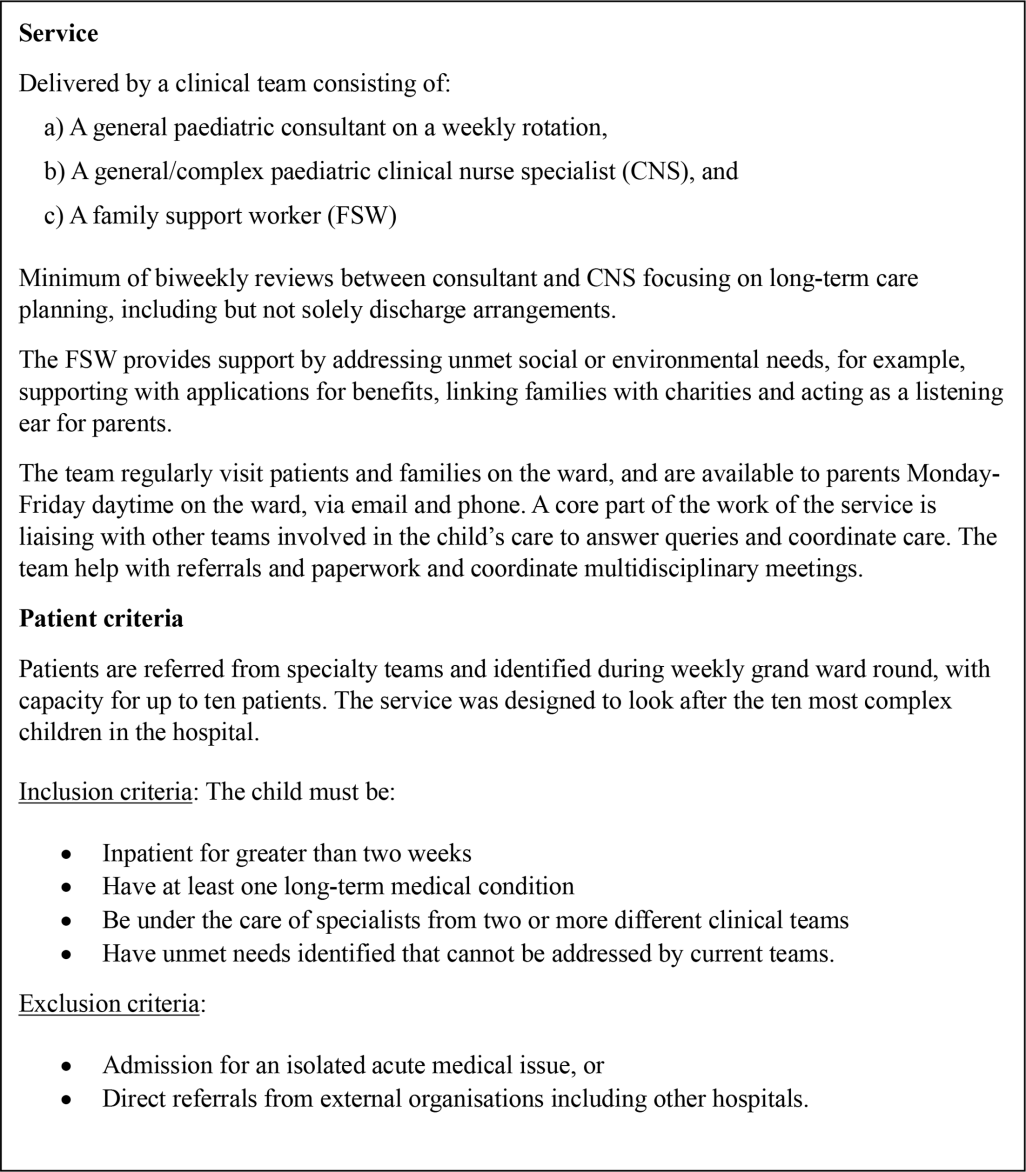
Description of the hospital-inpatient service for CMC. CMC, children with medical complexity.

The study setting was a specialised children’s hospital serving 2.4 million children in England. In 2019, the hospital introduced an inpatient service for CMC to improve family and interprofessional communication, coordination of care and continuity of care (see figure 1).

### Study design

This qualitative study consisted of semistructured interviews with parents and health and care professionals and was reported in accordance with the consolidated criteria for reporting qualitative research guidelines.^19^ The topic guides were developed based on published literature,^6 10 20 21^ discussions with paediatricians and reviewed after the first few interviews with minor changes made to the language and structure (see online supplemental file 1). The topic guide included a section on general perceptions of services and the wider system for CMC, before focusing on perceptions and experiences of the CMC hospital-inpatient service to help provide a system lens to the evaluation.

### Participant recruitment process

**Figure 2:**
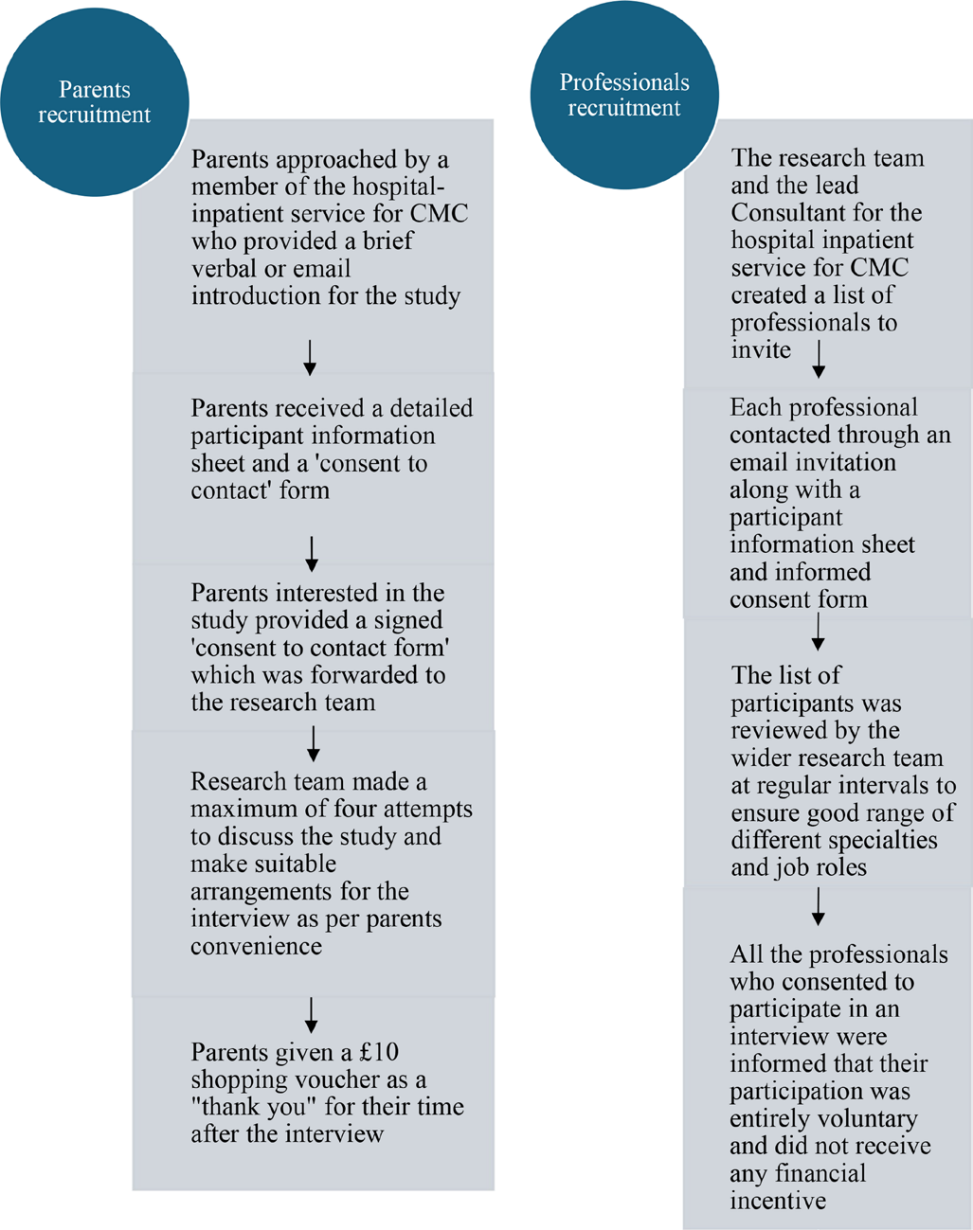
Participant recruitment process. CMC, children with medical complexity.

A purposive sample^22 23^ was drawn from two groups: (1) parents of CMC who had received care from the hospital-inpatient service and (2) professional stakeholders. We invited a mixture of professionals from different specialist teams who liaise with the service (including nursing staff, doctors and charity representatives both from within the hospital and from community services) and members of the hospital-inpatient service for CMC itself. We recruited parents of children currently under the service and those discharged from the service. Participants were recruited between December 2023 and May 2024. The recruitment process is described in figure 2.

### Patient and public involvement

The participant information sheet and informed consent form were piloted with parents of CMC not part of this study.

### Data management and analysis

One male postdoctoral researcher with experience in conducting qualitative research (SG) conducted interviews face-to-face or via Microsoft Teams. The interviewer was independent and not a part of the child’s care team. Interviews were recorded and transcribed verbatim (postinterview) and pseudonymised by the research team. Inductive thematic analysis was conducted (by SG and BP)^24^ using NVivo as follows.

1. Familiarisation: both authors reviewed and familiarised themselves with the transcripts, noting down initial thoughts and impressions.
2. Initial codes: each segment of interview relevant to ‘hospital-inpatient service for CMC’ was coded line by line.
3. Searching for themes: all codes were imported into a concept map in NVivo, and the two authors worked together to identify commonalities and patterns with particular attention to comparisons among parent perspectives, the core team delivering the service and wider professionals.
4. Reviewing themes: potential themes and subthemes were developed. The two authors met weekly to discuss the themes and generate a thematic map.
5. Define themes: a proposed thematic structure was presented to the wider research team, with revisions made to the names of the themes following the discussion.
6. Write up the findings: the findings were drafted and illustrated with carefully selected quotes.

## Results

### Participants

**Figure 3:**
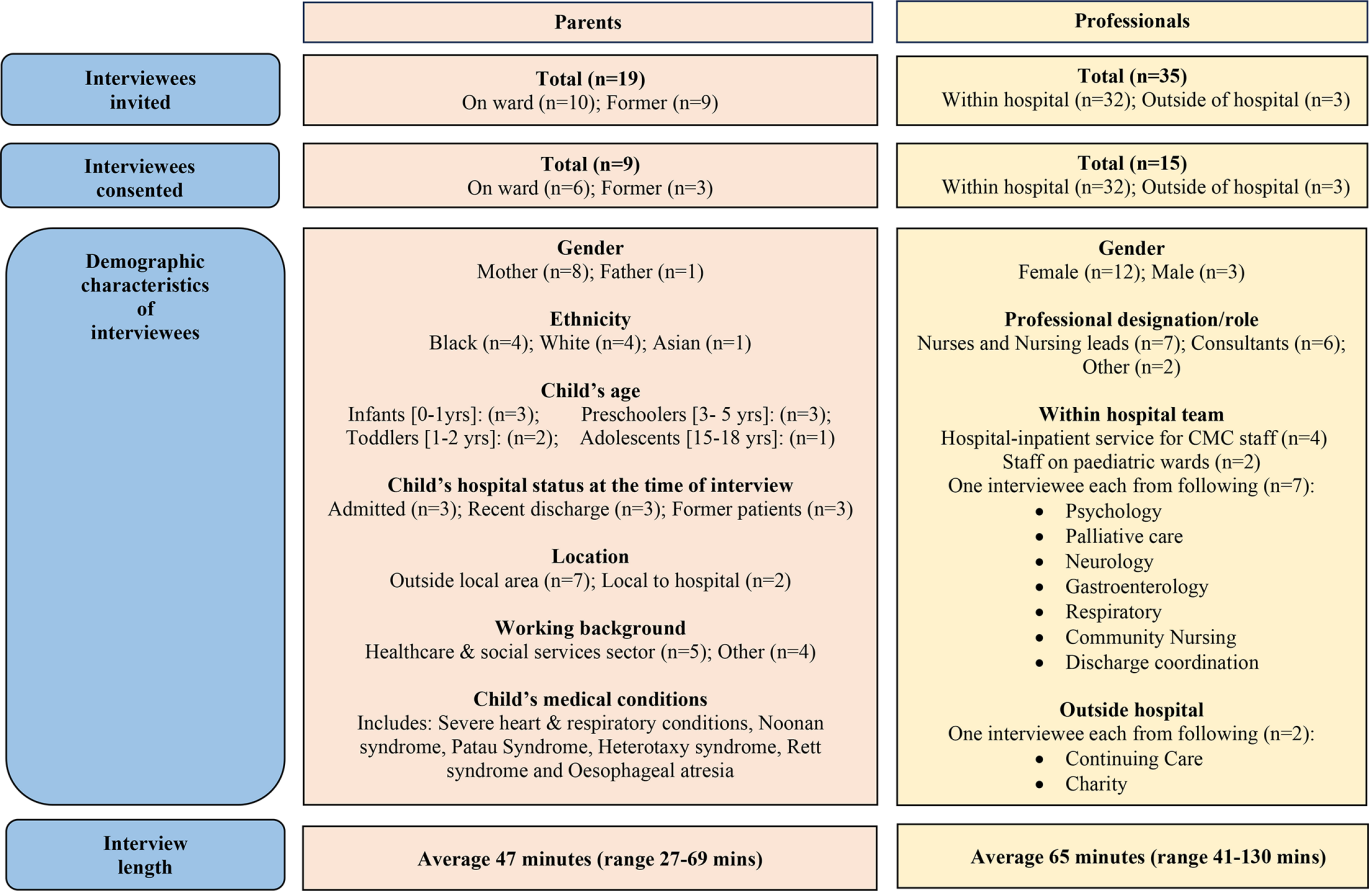
Characteristics of interviewees: parents and professionals. CMC, children with medical complexity.

The parents of 19 CMC were invited. Nine consented to participate and the remaining ten did not respond after four contacts by the research team. Similarly, among 35 professionals invited to participate, 15 consented, 2 declined and 18 did not respond. This resulted in a total sample of 24 participants whose characteristics are described in figure 3. The final sample included 6 parents of children currently cared for by the service and 3 parents of children who had since been discharged, and 4 professionals who currently work within the service and 11 professionals from other teams who liaise with the service or refer to the service. Quotes from professionals who worked within the hospital-inpatient service are marked with ‘CMC staff’.

### Themes

**Figure 4:**
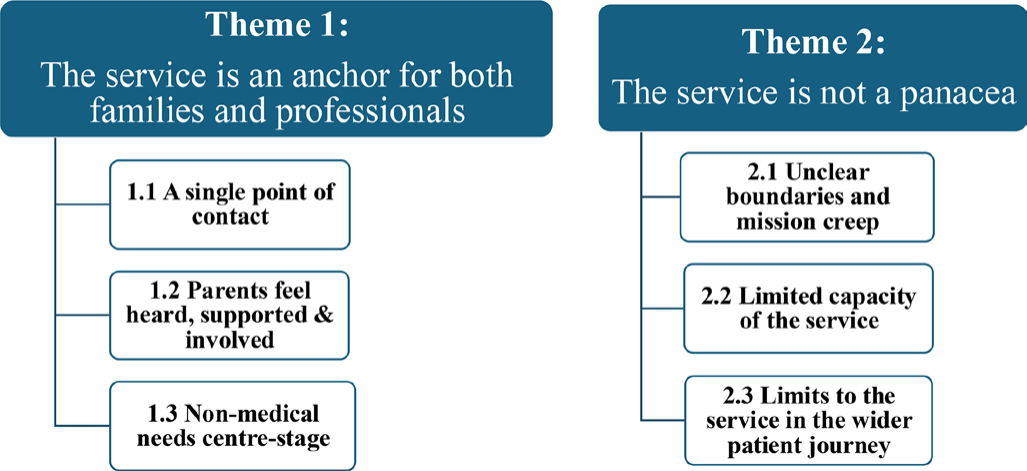
Overarching themes and subthemes.

Two overarching themes were developed. (1) *The service is an anchor for families and professionals’*. (ii) *The service is not a panacea* (figure 4).

#### Theme 1: the service is an anchor for families and professionals

The service served as a single point of contact to coordinate care, improve communication and ensure that the parent’s voice is heard. The service was considered to focus on the holistic needs of the child and families, pivotal for delivering family-centred care and highly valued by parents and professionals.

##### A single point of contact

The service provided a single point of contact for families and healthcare professionals, enhancing care navigation and leadership, promoting effective collaboration between clinical teams and ensuring that a clear and comprehensive care plan was established.

‘And then they (the hospital-inpatient service for CMC) came to me, and they introduced themselves. They said if I was happy for them to take care of our family, our package and when I ask what it was that about like in which way, she (service Consultant) explained me that, for example, if I would have any concern or any issues in getting. Because that is the thing (child) is under many medical teams, so for me, as a mom would be very difficult to be in touch with everyone and it is, I tried, and it is. So, she (service Consultant) said to me that they would be the ones, for example, updating everything what is being done? What needs to be done? Between hospitals? Being in touch with the medical teams on our behalf. And I straight away said, yes, please (Parent_7)’.

The service was widely recognised as a dedicated team playing an essential role in coordinating care, helping to ensure consistent decision-making among parents and professionals. This collaborative approach enabled everyone to work together towards clear shared objectives, ensuring that the right teams were involved and that *everyone’s kind of reading from the same hymn book* (Professional _2, CMC staff). The collaborative approach not only improved communication and coordination, but also *helped move things along* so that patients *do not get stuck* (Professional_15).

However, some parents expressed mixed feelings about the rotating nature of the consultants in the service regarding continuity in care.

A key strength of the service was its focus on coordinating discharge plans, which was valued by both parents and professionals. Discharge planning involved helping prepare parents, by building their confidence as carers and helping to make the necessary arrangements at home.

‘I think team kind of kept on top of the things that I needed to do in order for to prepare for her (child) to come home. So, every week they will kind of say, OK, this is what you need to do, this is what is outstanding. This is what she needs to be doing in order for her (child) to be discharged home. So that made it meant that by the time we came home, we actually felt a little bit confident to look after her (child)because we had had time and we had training and practice some of the things that we kind of would be doing at home while we were there, so yeah, there were, there were, there were really good with that. Just kind of preparing you for home and preparing the household (Parent_2)’.

Both parents and professionals felt that the service team provided *a holistic umbrella* and a more individualised approach to care for the child and family.

‘I think the biggest strengths they have is the relationships they build with families and their ability to think quite holistically about a family’s needs. I think because they are often taking a step back from focusing on one part of the body, they think they have more opportunities to think about the family as a whole, think about the child as a whole and their needs as a whole (Professional _3)’.

The service explained care plans to parents and relayed important information from other teams. The team fostered an environment where both parents and professionals felt comfortable approaching them, leading to better communication and ultimately enhancing the overall care experience. This approach alleviated stress for both parents and professionals. Professionals from other teams described feeling *relieved* when the service was involved.

##### Parents feel heard, supported and involved

Families felt that the service truly listens to their concerns and could be relied on. Parents appreciated the team advocating on their behalf and valued their excellent communication skills.

‘And I felt listened to, like they really took on what I was saying on board seriously. So, it always felt like your concerns were listened to, you felt like your concerns were valid. No one made you feel like you know what you are saying was not important or sound foolish or anything like that. So yeah, it was really good (Parent_3)’.

Some parents described past encounters with other medical teams where they felt they had not been listened to. However, they described the hospital-inpatient service for CMC as genuinely listening to their concerns: *they were the only ones who did listen and did take me seriously (Parent_6*). The service helped to empower families and advocated for parents by communicating their views and wishes to other teams involved in the child’s care. Their expert communication skills helped when conveying challenging news to families.

‘But I did appreciate that like she (CNS) was quite straightforward and frank with what the plan was going to be and what the difficulties and challenges that they were facing. Because I think sometimes there is a tendency for people to kind of sugarcoat things or to appease you, not to kind of upset you. Umm, but if you tell me things as they are, then I can kind of see how I want to manage it and how I feel about it. So that was quite, that was really, really good (Parent_2)’.

Families felt they could trust the team and that they were actively involved in decision-making about their child.

‘Whereas with the (hospital-inpatient service for CMC) team, you know, before anything is done for your child, you will have a discussion, you know, get my thoughts and my view. So you felt included in the care process regarding your child and the decision-making regarding your child. You know you did not feel like you were left out of the loop, or that you know there was something missing, or there is something you are not being told, and you know people are just acting shady, you know, yeah. So that is, that is the difference (Parent_3)’.

##### Holistic needs centre stage

Addressing the holistic needs of the family, such as housing, finances and parental well-being, is a fundamental role of the service. This support extends beyond signposting, playing a vital role in addressing housing and financial challenges that families appreciate.

‘We got help in terms of like filling out his disability living allowance forms and stuff, and that was a part of the (hospital-inpatient service for CMC) team as well. They helped us to do, we did not know anything about it, you know? So they have like, literally, we just signed the form basically, and they just helped us to fill the details, because we had so much going on to be thinking about what to put on the form and stuff, you know. And you know help with Council and rehousing in terms of like his long-term needs. Umm yeah and help with, financial help, where we can get financial help and stuff like that. So, it is been a really, really good, yeah. (Parent_3)’.

The service also supports the mental health and well-being of parents. Parents described how the team frequently asked parents how they were feeling, for example, checking whether parents had got enough sleep or offering a shoulder to cry on. The service acted as a *sponge* to absorb families’ stress.

‘So from the very, very beginning, they always been supporting and never judged, you know, never judged, if I would cry, they would say, cry if you need to cry, let me know if you need anything. If you need a hug, let me know, do not be afraid to speak out on behalf of your son because you know him better (Parent_7)’.

Both families and healthcare professionals viewed the Clinical Nurse Specialist (CNS) and Family Support Worker (FSW) as absolutely critical to the success of the service.

‘And particularly that a parent parental voice can be heard as well. From my experience that, that is done very effectively, particularly with the core team of the CNS and the FSW. The Consultant cover is incredibly helpful but would not work if it were not for the consistency of the core team (Professional _10)’.

### Theme 2: the service is not a panacea

It was clear that the service should not be seen as a panacea for fixing the wider provision of care for CMC. Participants described unclear boundaries around the role and reach of the service, challenges with limited capacity and concerns relating to a cliff edge postdischarge.

#### Unclear boundaries and mission creep

Professionals expressed uncertainty and variation in their perceptions of the aims and remit of the service. Many felt that the role of the service was to deliver holistic care and to coordinate care when children have multiple specialty teams involved. Some professionals felt that the service should have stronger boundaries and be clearer what was beyond their responsibility, and what the manifesto of the team is. Some perceived that the service was used by other teams to handle aspects of their roles that they would prefer to pass on to another team.

‘Once they (the hospital-inpatient service for CMC) become involved, all of a sudden, everyone else can, you know, effectively give up some of their responsibilities (Professional_10)’.

Other professionals felt that they never expected the service to take a lead on the child’s care who is under their specialty, rather expecting the service to perform an advisory role.

‘So rather than coming down and seeing our patient and kind of taking them on, are there ways in which we could kind of tap into that advice, around that learned experience around discharge planning and with complex care coordination, that kind of signposting, and pointing in the right direction (Professional_9)’.

Some professionals felt there had been some *mission creep* from the service, as they took on extra responsibilities.

‘So, I think that is, that is and it is expanded a little bit in terms of its viewpoint, particularly because we are seeing some children which are coming backwards and forwards and it has kind of moved slightly into this, this kind of more community facing, this children with recurrent admissions type group…(Professional_6, CMC staff)’.

In some cases, the service was used by other teams during conflict situations when the relationship between staff and a family had broken down, which was not an intended role of the service.

Some described a lack of clarity in the referral criteria (describing the criteria as *open to interpretation*). Others felt that the referral criteria had gradually changed over time. Others questioned whether the service was seeing the ‘right’ patients.

‘I also think the boundaries of which children the CMC service does or do not take umm, it can be a challenge both to hospital teams, individual disciplines within the hospital and can be a challenge to the CMC service themselves, umm yeah, as to, as to who they become involved with. And I think some clear guidelines that perhaps are published to the whole hospital might support that, umm in terms of, you know, if the child meets this, this, this and this criterion, then yes, we will take them or no, we would not, you know. And so a slightly clearer pathway might support that (Professional_10)’.

However, there was a contrary view from one professional who perceived that loose criteria actually assist the service to act as a network providing care for children in need. Additionally, there was a lack of clarity from some parents regarding involvement of the service postdischarge, but, otherwise, there were no concerns expressed by parents about unclear boundaries around the service.

‘Yeah, you see, that is the one thing I never really know if (child) was fully discharged by the CMC service. Because does the CMC service care stops when the hospitalisation ends? (Parent_9)’.

#### Limited capacity of the service

Concerns were raised about the service being overwhelmed, relating to limited capacity. Both parents and professionals acknowledged considerable stress in the team and high risk of burnout. The service was felt to be very reliant especially on the CNS, with no backup.

‘I did not hear anyone mention that, that there was someone other than […CNS] and they were like, OK, I will speak to […CNS] and I was like, oh, it is just (…CNS). There should be someone else to support her, you know, and whenever the doctor’s come in, it was always[…CNS] that was with doctors and no one else. And I am like…it must be…I am working in a mental hospital and I am like that must be quite a lot for her…on her own (Parent_1)’.

#### Limits to the service in the wider patient journey

Parents described the constant fight they experienced with the health and care system before the hospital-inpatient service for CMC was involved. Lack of a designated caregiver coordinator was a major concern, and this issue persists as many families still lack leadership in their postdischarge care.

‘They [parents] do not know where to start, and they often have children under multiple teams, and they do not seem to have somebody who is really leading them through that journey. Umm they kind of bounce between different teams, they get told different things (Professional _5)’.

Some contrary views were expressed from parents who perceived an overlap in the care received from the neonatal intensive care unit (NICU) who led the child’s care.

‘Umm when NICU, I think we were involved in the decision-making also and I also felt there was continuity of care uh, in NICU…And the surgical team, I think acted a little bit as the CMC service team while we were on NICU (Parent_9)’.

Parents and professionals described a stark drop-off in support following discharge for many (but not all) families, creating a sense of a cliff edge for some families. Concerns were raised about a lack of support in the community, including families struggling to get continuity of care from primary care services and a lack of expertise in the emergency department and from General Practitioners (GPs) to help with problems. Some professionals were concerned that families become reliant on the service and desire to seek support from the service after discharge. Participants described how some families continued to be in contact informally with the service team postdischarge.

‘It would be really nice to have somebody that you, just even rang you once, once you got out of hospital to see how things are going? Is there anything that we could do differently? Is there anything you need? Is everything going, OK? (Parent_6).

So the CMC service, today still the team, that I contact when I need to be in touch with the team in [the hospital]. And if I cannot get to them, then I just either email them or leave a voice message, and then someone from the team will come back to me and actually help me with that. So, it makes the process very, very, umm, how you say? Smooth, not stressful (Parent_7)’.

## Discussion

This study found that a hospital-inpatient service for CMC can be a valuable resource for families and professionals, serving as a single point of contact to manage care, enhance communication and ensure that parents' voices are heard during the hospital admission. The service was valued for its holistic focus on the needs of CMC and their families. The study highlights the importance of exploring how the service integrates with the wider health and care system. There were concerns related to a lack of support in the community postdischarge and issues with unclear roles and boundaries of the service.

### Capacity to deliver holistic care

Families of CMC face considerable challenges due to inadequacies in financial, practical and psychological support.^9^ There are individual, interpersonal and institutional barriers to health teams addressing the holistic needs of CMC and their families.^12^ Consistent with other studies on care coordinators and dedicated key workers,^10 25^ our data support the valuable role of professionals with dedicated time to prioritise the holistic needs of CMC and their families.^26^ The CNS and FSW were vital to this service because of their ability to coordinate care, advocate for families, listen attentively to their needs and assist with practical arrangements for discharge. This is similar to previous studies.^27 28^

### A system-wide approach to care coordination

Hospital-inpatient services for CMC can improve care coordination during hospital admissions; however, a comprehensive, system-wide approach to care coordination is needed to support children and families postdischarge. The transition from hospital to home can be complex and frustrating for parents^29^ and there is a need to ensure that families do not feel abandoned by episode-based models of care like the one in this study.^12^

Those seeking to set up similar services for CMC need to see the new service through the eyes of families and the wider patient journey, considering how to integrate with community and primary care services to improve the care of CMC beyond the hospital admission and ensuring sufficient resources to do so consistently. Other countries have explored care coordination models, which work across hospital and community care but there is mixed evidence on their effectiveness.^30^,^32^ In the UK, the majority of paediatric expertise is concentrated in secondary and tertiary care settings.^12 33^ Meanwhile, the families of CMC often report that GPs in primary care are unable to meet their needs.^8 9 34^ A large amount of care for CMC is provided in non-traditional care settings (eg, home and school)^35^: there is a need to explore the models of care, which are feasible within the UK context, which can help coordinate care across these settings. This may be an area for integrated care systems to assist with, as regional organisations recently implemented in England and expected to bring health and care together to work more effectively at population level.^36^

### Service boundaries and mission creep

When designing services, it is critical to identify which patients to focus resources on (referral and acceptance criteria), and where the service can add most value to the wider system. Critically, the service does not have the capacity to look after all patients who would meet the definition of medical complexity: the service only has capacity to look after the ten most complex. Defining the criteria for most complex is an ongoing challenge for the service. The introduction of any new service can disrupt the existing services or workflow. It is important to regularly re-evaluate the impact of a service not just on patient and family outcomes but also on the impact on other services: there is a risk of duplicating work of other teams. This study highlights the importance of well-defined aims and responsibilities, which are clearly communicated to other teams and services.

### Implications for research

Services for CMC exist in other hospitals in the UK and internationally but there has been limited evaluation.^12^ Research is needed to evaluate and compare models in different settings to inform policy nationally and internationally. There is a need to explore quantitative outcomes from the health system perspective (eg, ‘length of hospital stay’) and outcomes for children and families (eg, parental mental health and measures of care coordination or family-centred care).^10 35^ Assessment of the effectiveness of care models is currently hampered by a lack of standardised outcomes and population definitions for CMC.^31 35 37^

### Strengths and limitations

A strength of the sample of parents was that it included families from a range of different ethnicities, child’s hospital admission status and medical conditions. Parents were invited by a member of the hospital-inpatient service, and therefore, there is a possibility of gatekeeping and bias in the sample. In addition, participants who could not read or speak English were excluded and there was only one father in the sample. We were not able to explore the experiences postdischarge of some of the parents, as six were still in hospital under the care of the service and this was not a longitudinal study. Among the professional group, we recruited participants from a range of different specialties and jobs roles; however, the sample did not include many professionals from outside of the hospital setting, so we were unable to explore postdischarge care in much detail. Some of the professionals had extensive direct experiences with the service, for example, staff who worked within the service, whereas others had less direct experience and reflected more on their perceptions of the service, for example, some specialist consultants referring to the service. It is not possible to comment on whether participants who did not respond to the study invitation differed from those who participated.

## Conclusion

A hospital-inpatient service for CMC can improve care coordination and help to build strong relationships with parents so that they feel listened to and supported. Services need to have clear boundaries and need to be well integrated with services in the community posthospital discharge. Future interventions should ensure a system-wide approach to development, integration and evaluation.

## supplementary material

10.1136/bmjpo-2024-003101 online supplemental file 1

## Data Availability

No data are available.
