# Supplementary material for: Qualitative evaluation of a hospital-inpatient service for children with medical complexity
Source: BMJ Paediatr Open. 2025 Mar 5;9(1):e003101. doi: 10.1136/bmjpo-2024-003101 (PMC11883592; doi:10.1136/bmjpo-2024-003101)
Supplement: online supplemental file 1 [file bmjpo-9-1-s001.pdf]

Supplementary File 1: Topic guides

Interview Topic Guide and Prompts for Parents/Carers

Part 1: Researcher to introduce themselves and the interview plan and background.

1. **Introduce yourself.**

2. **Aim of the study**

   The aim of this study is to explore the views of key stakeholders on integration of care for children with special care needs.

3. **Why have you been invited?**

We want to hear about your experiences; your views are vital to shaping the study.

4. **Organization and funding**

   The study is being organised and funded by the National Institute for Health and Care Research.

5. **About the interview:**

   i. Will last for 45-60 min.

   ii. It will explore your views and experiences

   iii. You are free to stop the interview at any time, and you don't have to answer any questions you don't want to answer.

   iv. We will be recording interview for analysis.

   v. Only the research team has access to the interview recording

   vi. Our reporting will be completely anonymised.

vii. Findings from interviews will be used to reshape the service

viii. Thank you for consenting to take part in this interview

ix. If you have any questions – we can answer them now before we start.

Part 2. Interview with parent/caregiver

1) Individuals and their role.

a) Can you tell me about yourself and your child/ren?
(Parent [mother/father] or caregiver, family members, child's disability/condition, when got the diagnosis/news about it)

2) Identify and address medical and psychosocial needs.

a) What is a typical day like? (Family, who live with, nearby area, home set-up, work/school/responsibilities, other activities, interaction with the community)

b) In general, how are things for you and your child/ren currently, and over the last few years? (Quality of life and wellbeing for you and child, any changes to circumstances or significant events)

c) How are things since referral to CMC service? (explore views on overall service and also individual role i.e. Consultant, CNS and FSW)

3) Perceptions of key stakeholders on CHSCN and family satisfaction with care, and care integration, including equity of access and how this is impacted by integration.

a) What is your experience of being listened to and involved in decision making and planning? If relevant, can you describe your experience of care and support for your child before the CMC service?

b) If relevant, how did your experience of care and support (before CMC service) impact your family? (impact on your child, yourself, other family members; in terms of: wellbeing and health of family, school/work/responsibilities, friends and support, ability to live as would like to).

c) Are there things that you have found to be particularly difficult? (number of appointments and burden of healthcare coordination, on time and wellbeing; missing

work/family responsibilities; able to work if wish to; stress; engagement/relationships/conflict with care providers)

d) How have you dealt with these challenges? (Strategies employed to mitigate the impacts, other people/factors that might have been involved)

4) Perception of key stakeholders on the impact of service integration on CSHCN and family, including care coordination, communication and continuity.

a) Understanding and expectations:
   i) When was your child referred to CMC service?

   ii) What was your understanding and expectations of CMC service when your child's care was started?

b) What are/were the positive or useful aspects of the 'CMC service'?

c) What are/were the difficult or unhelpful aspects of the 'CMC service' and the inpatient experience?

d) What expectations were met and not met?

e) Have you used the navigation packs? What were helpful and unhelpful aspects?

f) Have you used the electronic patient portal (MyChart)? What were helpful and unhelpful aspects?

g) Do you have any suggestions on how services should be changed?

5) Other comments: Is there anything else you would like to share about your experience with being a parent of a child with special healthcare needs OR this service?

6) Closure: Thank participants and end session.

Interview Topic Guide and Prompts for Staff and Professionals

Part 1: Researcher to introduce themselves and the interview plan and background.

6. **Introduce yourself.**

7. **Aim of the study:**

   The aim of this study is to explore the views of key stakeholders on integration of care for children with special care needs.

8. **Why have you been invited?**

   We want to hear about your experiences; your views are vital to the study.

9. **Organization and funding**

   The study is being organised and funded by National Institute for Health and Care Research.

10. **About the interview:**

    i. Will last for 45-60 min.

    ii. It will explore your views and experiences

    iii. You are free to stop the interview at any time, and you don't have to answer any questions you don't want to answer.

    iv. We will be recording interview for analysis.

    v. Only the research team has access to the interview recording

    vi. Our reporting will be completely pseudonymised.

    vii. Findings from interviews will be used to reshape the service

    viii. Thank you for consenting to take part in this interview

    ix. If you have any questions – we can answer them now before we start.

Part 2. Interview with staff/professionals

7) **Individuals and their role.**

   a) Can you tell me about yourself and your role regarding CSHCN? (Training/years of experience, professional background)

8) **Identify and address medical and psychosocial needs.**

   a) In general, how have things been for CSHCN and their families in South London over the last few years?

   b) In general, how have things been for CSHCN and their families in Evelina?

   c) What is your perception of CSHCN and their families, thinking about their quality of life, wellbeing, and inclusion in the community in South London

9) **Perceptions of key stakeholders on CHSCN and family satisfaction with care, and care integration, including equity of access and how this is impacted by integration.**

   a) What is your experience of listening to parents/patients? How does this work day-to-day or week-to-week? What makes this easier? What makes it harder?

   b) What are your views of the integration of care and support currently available for CSHCN in South London? (Prompts: Family satisfaction with care and care integration; Engagement/conflict between families and services; Equity of access and how impacted by integration)

   c) What are the positive and negative aspects of working with CSHCN and their families? (Staff morale, staff retention; Career plans)

   d) How has this changed or not changed since the introduction of CMC service?

10) **Perception of key stakeholders on the impact of service integration on CSHCN and family, including care coordination, communication and continuity.**

   a) Understanding and Expectation:
      i) Explain your role with respect to CMC service service? (are you a part of the CMC service team or have you referred patients to CMC service)

      ii) What is your understanding and expectations of CMC service service? (explore about Consultant, CNS and FSW separately)

   b) What are the positive or useful aspects of the CMC service?

(explore in terms of coordination, communication and continuity)

c) What were the difficult or unhelpful aspects of the CMC service?
(see what staff/professionals say and also probe in terms of coordination, communication and continuity)

d) What expectations were met and not met?

e) How could this be improved or what could have been done differently?

f) What are your views on the navigation packs? What are helpful and unhelpful aspects?

g) How have you used the electronic patient portal (MyChart)? What were helpful and unhelpful aspects?

h) Do you have any suggestions on how this service should be changed?

11) **Other comments:** Is there anything else you would like to share about your experience or any comment on the service, and other elements of care integration?

Closure: Thank participants and end session.
