# Supplementary material for: Qualitative evaluation of a hospital-inpatient service for children with medical complexity
Source: BMJ Paediatr Open. 2025 Mar 5;9(1):e003101. doi: 10.1136/bmjpo-2024-003101 (PMC11883592; doi:10.1136/bmjpo-2024-003101)
Supplement: Supplementary file 2 [file bmjpo-2024-003101.reviewer_comments.pdf]

PEER REVIEW HISTORY

BMJ Paediatrics Open publishes all reviews undertaken for accepted manuscripts. Reviewers are asked to complete a checklist review form and are provided with free text boxes to elaborate on their assessment. These free text comments are reproduced below.

ARTICLE DETAILS

**TITLE (PROVISIONAL)**	A qualitative evaluation of a hospital-inpatient service for children with medical complexity
**AUTHORS**	Ghotane, Swapnil; Page, Bethan; Ramachandran, Rohanna; Wolfe, Ingrid; Fraser, Lorna

VERSION 1 - REVIEW

**REVIEWER NAME**	***Charlotte Castor***
**REVIEWER AFFILIATION**	Lund Univ
**REVIEWER CONFLICT OF INTEREST**	Not applicable
**DATE REVIEW RETURNED**	06-Nov-2024

**GENERAL COMMENTS**	Overall this is a well written manuscript describing a qualitative evaluation of a hospital-based inpatient service as an addition to the usual care for children with complex medical conditions. This is in line with early integration of palliative care and a palliative care approach even though the authors chose note to use these phrasings. The manuscript brings up important issues for concern when implementing such a service and I would like to give the authors credit for publishing an evaluation not fully supporting the intervention. Below you will find some suggestions which I believe would further strengthen the manuscript.<br>ABSTRACT<br>In the last sentence of the findings (although in the main manuscript called results) the authors state that "some professionals and families" describe a cliff-edge post hospital discharge. Is there a specific need to point out that it was some and not all? Did some explicitly express the opposite or was it that there was a diversity in feelings for this period of illness? I suspect that many of the experiences are not described by all participants.<br>In the conclusions you use both "hospital inpatient service" and "hospital-based inpatient service" is there a difference?<br>The authors state under How this study might affect research, practice, or policy "that a dedicated inpatient holistic service cannot address gaps in the wider system." This seems to be a definite conclusion if drawn from one qualitative study. This might have been the case in this particular study and it certainly indicates challenges but are the authors certain that this is not possible at all? Could there for example be adjustments of the service to better address border confusion and wider system issues?<br><br>INTRODUCTION<br>Clear and well-structured

	AIM<br>Is there a need and place for both experiences and perceptions in the aim? If yes did the authors distinguish between these during analysis? Are they meant to complete each other? Could this be elaborated on in the design or discussion<br><br>METHODS<br>Figure 1:<br>Is it an overview or a description? Authors use both words.<br>It is unclear to me what is meant by “more than one specialist”. More than one physician, from different teams?<br>Inclusion criteria is ONE long-term medical condition. Does this mean children with more than one are excluded from the service?<br>I would have liked a more detailed description of how this service is carried out and actually interact with other parts of healthcare as well as the family. Would it be possible to add an example? For example the meaning of theme 1.1 is difficult to comprehend without knowing how this service interact with families. Were there, for example, a telephone service available and if yes 24/7 or daytime and with whom?<br><br>Supplementary file 1. Topic guides:<br>Extensive with several questions related to life and care before CMC. For professional Caregivers CMC is mentioned for the first time in question 9d… It seems as if there must be much data not included in this analysis. Should this possible be addressed under analysis<br><br>In the paragraph on design authors state that the interview guide was reviewed. Did this review lead to any revisions?<br><br>A more thorough description of participants and recruitment strategy would be valuable in order to understand the findings. See comments in the results section<br><br>RESULTS<br>I would have preferred the information on eligible participants in the methods section and I was glad to finally find it in the results.<br>In the methods section the authors describe a purposeful sampling strategy however the one described here seems to be more of a convenience sapling. Would it be possible to describe if the once participating differed or not from the once not participating. Further, it is unclear to me whether or not participants from the CMC service are also participants. This should be described more clearly. Are they evaluating themselves?<br><br>Quote Parent 7 page 8 line 153 is a bit difficult to read. This might be the way the parent expressed her/his self however it seems there are words missing. Other than that, an informative quote.<br><br>Please adjust for conformity of phrasing trough out the manuscript: hospital-inpatient service/the team/service team/CMS-service/ the service… Further in figure 2 child’s clinical team and clinical care teams are mentioned and in figure 3 also Core clinical team

	delivering service is mentioned. How are these connected or different from each other?<br><br>Theme 2.2 is relatively long and also with heavy overweight of professional's perspectives. Especially for this subtheme it would be relevant to know which quotes comes from professionals working in a service team and which comes from other teams<br><br>Phrasing of 2.3 seems to differ from the other themes and subthemes. Can it be rephrased by simply withdraw "the"?<br><br>DISCUSSION<br>The design, especially the sampling, brings both strengths and limitations. Trustworthiness of the study would increase if the authors elaborated on this.

**REVIEWER NAME**	***Maha Atout***
**REVIEWER AFFILIATION**	Philadelphia University Faculty of Nursing
**REVIEWER CONFLICT OF INTEREST**	N/A
**DATE REVIEW RETURNED**	22-Dec-2024

**GENERAL COMMENTS**	Thank you very much for inviting me to review this fascinating and important study. The study's writing is commendable. I have two minor suggestions.<br><br>Renew some old references.<br>I think that the inclusion criteria of the CMC are not congruent with its definition. For instance, explain how you incorporated technology dependence into the inclusion criteria. Moreover, is it important to include this inclusion criteria item:<br>Have parents identified unmet needs that current teams are unable to address?

VERSION 1 – AUTHOR RESPONSE

Comment	Response
Reviewer 1	
Overall this is a well written manuscript describing a qualitative evaluation of a hospital-based inpatient service as an addition to the usual care for children with complex medical conditions. This is in line with early integration of palliative care and a palliative care approach even though the authors chose note to use these phrasings. The manuscript brings up important issues for concern when implementing such a service and I would like to give the authors credit for publishing an evaluation not fully supporting the intervention. Below you will find some suggestions which I believe would further strengthen the manuscript.	Thank you for the positive feedback

ABSTRACT<br>In the last sentence of the findings (although in the main manuscript called results) the authors state that “some professionals and families” describe a cliff-edge post hospital discharge. Is there a specific need to point out that it was some and not all? Did some explicitly express the opposite or was it that there was a diversity in feelings for this period of illness? I suspect that many of the experiences are not described by all participants.	Thank you for this helpful point for clarification. Of those participants (professionals and parents) who did comment on the post discharge experience, the majority expressed concerns about a lack of support with some suggesting that the hospital-inpatient service for CMC should follow up with families post discharge. Conversely one parent described feeling well supported by community teams post discharge. We have clarified this in the abstract (“*Professionals and families described a cliff-edge for many families post hospital discharge”*) and in theme 2.3 in the results.
In the conclusions you use both “hospital inpatient service” and “hospital-based inpatient service” is there a difference?	We have revised the terminology to “hospital inpatient service” and removed the term “hospital-based inpatient service”
The authors state under How this study might affect research, practice, or policy “that a dedicated inpatient holistic service cannot address gaps in the wider system.” This seems to be a definite conclusion if drawn from one qualitative study. This might have been the case in this particular study and it certainly indicates challenges but are the authors certain that this is not possible at all? Could there for example be adjustments of the service to better address border confusion and wider system issues?	We have reworded this sentence to improve clarity: “*there are significant gaps in the wider system (e.g. community and social care) which it [the hospital-inpatient service for CMC] has limited capacity to address”*
INTRODUCTION<br>Clear and well-structured	Thank you
AIM<br>Is there a need and place for both experiences and perceptions in the aim? If yes did the authors distinguish between these during analysis? Are they meant to complete each other? Could this be elaborated on in the design or discussion	We have included both terms in the aim to reflect the fact that some participants (e.g. the parents and some of the professionals) have lots of direct experiences of the service, whereas some professionals had less direct experience with the service but have views on what they think the service does or should do, and therefore their data was more ‘perceptions’, e.g. they referred into the service but had limited direct experience interacting with the service. We have added a sentence to the discussion to elaborate on this in the manuscript: “*Some of the professionals had extensive direct experiences with the service, e.g. staff who worked within the service or staff on the paediatrics wards, whereas others had less direct experience and reflected more on their perceptions of the service, e.g. some specialist consultants referring into the service.”*
Figure 1:<br>Is it an overview or a description? Authors use both words.	Thank you for spotting this. We have edited to use the word ‘description’ and removed the word ‘overview’.
It is unclear to me what is meant by “more than one specialist”. More than one physician, from different teams?	We have edited Figure 1 to clarify that this is *“Be under the care of specialists from two or more different clinical teams”*

Inclusion criteria is ONE long-term medical condition. Does this mean children with more than one are excluded from the service?	Thank you for spotting this. We have clarified this and it now says "*have at least one long-term medical condition*".
I would have liked a more detailed description of how this service is carried out and actually interact with other parts of healthcare as well as the family. Would it be possible to add an example? For example the meaning of theme 1.1 is difficult to comprehend without knowing how this service interact with families. Were there, for example, a telephone service available and if yes 24/7 or daytime and with whom?	Thank you for this helpful suggestion. We have added additional information to Figure 1 to provide more details about the service:<br><br>"**Service**<br>*Delivered by a clinical team consisting of:*<br>*a) A general paediatric consultant on a weekly rotation,*<br>*b) A general/complex paediatric clinical nurse specialist (CNS), and*<br>*c) A family support worker (FSW)*<br><br>*Minimum of biweekly reviews between consultant and CNS focusing on long-term care planning, including but not solely discharge arrangements.*<br><br>*The FSW provides support by addressing unmet social or environmental needs, for example, supporting with applications for benefits, linking families with charities and acting as a listening ear for parents.*<br><br>*The team regularly visit patients and families on the ward, and are available to parents Monday-Friday daytime on the ward, via email and phone. A core part of the work of the service is liaising with other teams involved in the child's care to answer queries and coordinate care. The team help with referrals and paperwork and coordinate multidisciplinary meetings."*
Supplementary file 1. Topic guides:<br>Extensive with several questions related to life and care before CMC. For professional Caregivers CMC is mentioned for the first time in question 9d… It seems as if there must be much data not included in this analysis. Should this possible be addressed under analysis	The first part of the topic guides focus on understanding the job role of professionals (professional guide)/ the needs of the child and parents (parent guide). The next section focuses on general perceptions of services and the wider system for children with medical complexity, before focusing on perceptions and experiences of the CMC hospital inpatient service. The earlier sections are helpful context for understanding the role of the CMC hospital inpatient service in the wider system which is critical for the results. There is some data on the wider system from the interviews which is not included directly in the analysis, but it is critical context for understanding how the service fits into the wider systems. The system lens is a key strength of this paper.<br><br>The following sentence has been added to the methods:<br>"*The topic guide included a section on general perceptions of services and the wider system for*

	children with medical complexity, before focusing on perceptions and experiences of the CMC hospital inpatient service to help provide a system lens to the evaluation."
In the paragraph on design authors state that the interview guide was reviewed. Did this review lead to any revisions?	Minor changes to the language and structure of the interview guide were made. This has been clarified in the methods.
A more thorough description of participants and recruitment strategy would be valuable in order to understand the findings. See comments in the results section	See below for the additions we have made to describe the recruitment strategy and description of participants.
RESULTS<br>I would have preferred the information on eligible participants in the methods section and I was glad to finally find it in the results.	We have added some additional information on recruiting the sample to the methods (see below). We have left the information on the final sample at the start of the results, as we see this as part of the results.
In the methods section the authors describe a purposeful sampling strategy however the one described here seems to be more of a convenience sapling. Would it be possible to describe if the once participating differed or not from the once not participating. Further, it is unclear to me whether or not participants from the CMC service are also participants. This should be described more clearly. Are they evaluating themselves?	We have added some additional material on the sample to the methods and results section to supplement the information on recruitment of participants and demographics in Figures 2 and 3.<br><br>Methods- participant recruitment process<br><br>*"We invited a mixture of professionals from different specialist teams who liaise with the service (including nursing staff, doctors and charity representatives both from within the hospital and from community services), and members of the core hospital-inpatient service itself. We recruited parents of children currently under the service and those discharged from the service."*<br><br>Results- participants<br><br>"*The final sample include 6 parents of children currently cared for by the service and three parents of children who had since been discharged, and four professionals who currently work within the service and 11 professionals from other teams who liaise with the service or refer into the service."*<br><br>It is not possible to describe if the participants participating differed from those not participating. This has been added to the limitations.<br><br>*"It is not possible to comment on whether participants who did not respond to the study invitation differed from those who participated."*
Quote Parent 7 page 8 line 153 is a bit difficult to read. This might be the way the parent expressed her/his self however it seems there	We have added some additional text in square brackets to help make this quote clearer. Some of the participants did not have English as the first language so sometimes the language is a little

are words missing. Other than that, an informative quote.	unclear. We have used the direct words of the participants and added material in square brackets to make things clearer.
Please adjust for conformity of phrasing trough out the manuscript: hospital-inpatient service/the team/service team/CMS-service/ the service…	We have edited to refer to the service as the "hospital-inpatient service for CMC".
Further in figure 2 child's clinical team and clinical care teams are mentioned and in figure 3 also Core clinical team delivering service is mentioned. How are these connected or different from each other?	We have edited Figure 2 and 3 to make it clearer and the language more consistent. Both now refer to staff working in the "hospital-inpatient service for CMC".
Theme 2.2 is relatively long and also with heavy overweight of professional's perspectives. Especially for this subtheme it would be relevant to know which quotes comes from professionals working in a service team and which comes from other teams	We think the reviewer is referring to theme 2.1 and not 2.2 (which is the longer subtheme). We have added a note to any quotes from professionals who worked within the hospital-inpatient service for CMC, marking them as CMC staff (there are 4 of these participants). Only 2 quotes in the results come from this group of professionals, with one in subtheme 2.1.<br>Nearly all the data from this subtheme does come from professionals, we have added a sentence to comment on the lack of data from parents.<br>We have removed a few quotes and sentences to shorten this subtheme.
Phrasing of 2.3 seems to differ from the other themes and subthemes. Can it be rephrased by simply withdraw "the"?	We have withdrawn the word "the" from the title of the subtheme, and amended Figure 4 (diagram of overarching themes and subthemes) accordingly.
DISCUSSION<br>The design, especially the sampling, brings both strengths and limitations. Trustworthiness of the study would increase if the authors elaborated on this.	We have added additional material to the strengths and limitations section to discuss the sampling and design in more detail.<br><br>e.g. "*We were not able to explore the experiences post discharge of some of the parents, as six were still in hospital under the care of the service and this was not a longitudinal study"*<br>*"Some of the professionals had extensive direct experiences with the service, e.g. staff who worked within the service, whereas others had less direct experience and reflected more on their perceptions of the service, e.g. specialist consultants referring into the service. It is not possible to comment on whether participants who did not respond to the study invitation differed from those who participated*."
Reviewer 2	
Thank you very much for inviting me to review this fascinating and important study. The study's writing is commendable.	Thank you for the positive feedback
I have two minor suggestions.<br>Renew some old references.	We have updated a few of the references. 17 of the references have been published since 2020 with 6 published in 2024.
I think that the inclusion criteria of the CMC are not congruent with its definition. For instance, explain how you incorporated technology	The inclusion criteria for the service is a subset of children with medical complexity. The design of the service as outlined in Figure 1 is to look after the 10

dependence into the inclusion criteria. Moreover, is it important to include this inclusion criteria item:<br>Have parents identified unmet needs that current teams are unable to address?	most complex children in the hospital, not all children with medical complexity The hospital looks after a large number of children with medical complexity who are technology dependent, but the service only has capacity for 10 patients. We have made this clearer in Figure 1 and added a sentence to the discussion under service boundaries and mission creep to discuss this.<br><br>*"Critically the service does not have capacity to look after all patients who would meet the definition of medical complexity: the service only has capacity to look after the 10 most complex. Defining the criteria for most complex is an ongoing challenge for the service."*<br>The decision to refer a patient to the service is made by clinicians and there is not currently an opportunity for parents to identify unmet needs and refer themselves to the service.

`

VERSION 2 – REVIEW

**REVIEWER NAME**	***Charlotte Castor***
**REVIEWER AFFILIATION**	Lund Univ
**REVIEWER CONFLICT OF INTEREST**	NA
**DATE REVIEW RETURNED**	16-Feb-2025

**GENERAL COMMENTS**	The manuscripts has gained clarity. The authors have given my comments respectful consideration and revised accordingly when adequate.

**REVIEWER NAME**	***Maha Atout***
**REVIEWER AFFILIATION**	Philadelphia University Faculty of Nursing
**REVIEWER CONFLICT OF INTEREST**	N/A
**DATE REVIEW RETURNED**	04-Feb-2025

**GENERAL COMMENTS**	Thank you for inviting me to review the revised version of the manuscript. I think that the authors addressed the required revision appropriately.<br>Well done!
